# Hydroxocobalamin for the treatment of vasoplegia after lung transplantation: A case series

**DOI:** 10.1016/j.jhlto.2024.100189

**Published:** 2024-11-29

**Authors:** Anh Nguyen, Rima Bouajram, Marek Brzezinski, Sahand Hassanipour, David Gordon, Binh Trinh, Tobias Deuse, Aida Venado, Steve Hays, Jonathan Singer, Jasleen Kukreja

**Affiliations:** a Baylor College of Medicine, Houston, Texas; b University of California San Francisco, San Francisco, California

**Keywords:** hydroxocobalamin, vasoplegia, lung transplantation, mean arterial pressure, cummulative vasopressor index

## Abstract

**Background:**

The use of hydroxocobalamin following lung transplantation has not been previously reported. We present a series of 3 cases where hydroxocobalamin was used to treat postoperative vasoplegia.

**Methods:**

We conducted a single-center, retrospective review of lung transplantation recipients from January 2016 to December 2020. We used cumulative vasopressor index to standardize vasopressor dose administered and mean arterial pressure at 2- and 24-hour time-points following hydroxocobalamin administration to assess treatment effectiveness.

**Results:**

We identified 3 male patients aged 49 to 62, with lung allocation scores between 89.9 and 90.6, requiring extracorporeal membrane oxygenation support (pre- and post-transplant for 5, 5, 9 and 8, 2, 2 days, respectively). Each patient received hydroxocobalamin 5,000 mg infused over 15 minutes, with patient #3 receiving an additional 6 doses over the subsequent 4 days. At the 2-hour time-point, mean arterial pressure increased in all patients (+11%, +17%, and +13%, respectively), although cumulative vasopressor indexes were inconsistent. At 24 hours, patients #1 and #2 demonstrated a marked increase in mean arterial pressure (36% and 23%, respectively) and a decrease in cumulative vasopressor index, while patient #3 displayed stable with slight reduction in cumulative vasopressor index and mean arterial pressure values. No allergic reactions were observed. Patient #3 developed methemoglobinemia and a medication-related false increase in triglycerides. All 3 patients were discharged home.

**Conclusions:**

Hydroxocobalamin may be a valuable adjunct in managing refractory vasoplegia following lung transplantation.

## Background

Vasoplegia, a form of vasodilatory shock characterized by low mean arterial pressure (MAP) and systemic vascular resistance refractory to standard vasopressors,^1^ can occur in up to 20%^2^ of patients undergoing cardiac surgery with cardiopulmonary bypass. Although various treatment options are available, managing vasoplegia can be challenging.^3^, ^4^, ^5^, ^6^, ^7^, ^8^, ^9^ Hydroxocobalamin (Cyanokit, Pfizer, Columbia, MD), an activated form of vitamin B12, has recently emerged as an alternative for treating vasoplegia in cardiac surgery and liver transplantation.^10^, ^11^, ^12^, ^13^, ^14^, ^15^, ^16^, ^17^ However, its application in lung transplantation (LTx) remains underexplored. Hydroxocobalamin is thought to work by inhibiting the synthase and binding of nitric oxide.^18^, ^19^ Here, we report our initial experience with hydroxocobalamin in 3 patients who developed severe, refractory vasoplegia following bilateral LTx.

## Methods

We conducted a single-center retrospective review of LTx recipients from January 2016 to December 2020. The study was reviewed and approved by our institutional Internal Review Board. Patients with refractory vasoplegia treated with hydroxocobalamin were identified using an electronic pharmacy database. Demographic and clinical data were extracted from electronic medical records. To assess the effectiveness of hydroxocobalamin, we monitored the cumulative vasopressor index (CVI) and mean arterial pressure (MAP) up to 24 hours postadministration. CVI, a well-established metric, for standardizing vasopressor dosing, is detailed in Table 1.^20^ Adverse effects and laboratory abnormalities associated with hydroxocobalamin therapy were also evaluated.

**Table 1:** Cumulative Vasopressor Index Calculation Based on Doses of Vasoactive Agents

Vasoactive agent	Dose range			
	1 point	2 points	3 points	4 points
Dopamine	0 < dose ≤ 5	5 < dose ≤ 10	10 < dose ≤ 15	>15
Epinephrine	–	0 < dose ≤ 0.05	0.05 < dose ≤ 0.1	>0.1
Norepinephrine	–	0 < dose ≤ 0.05	0.05 < dose ≤ 0.1	>0.1
Phenylephrine	–	0 < dose ≤ 0.4	0.4 < dose ≤ 0.8	>0.8
Vasopressin	–	–	–	Any dose

All doses are in mcg/kg/min except vasopressin, which is U/min.

## Result

Over the 5-year study period, our center performed 316 lung transplants, with 25% requiring extracorporeal membrane oxygenation (ECMO) as bridge-to-transplant and 19% requiring ECMO as bridge-to-recovery post-transplant. Patient characteristics are detailed in Table 2. We identified 3 male patients, aged 62, 61, and 49, all with interstitial lung disease (ILD) who developed rapidly progressive hypoxemic respiratory failure accompanied by severe right and/or biventricular dysfunction necessitating the use of dual lumen single site venovenous (VV) or femoral venoarterial (fVA) ECMO support as a bridge to lung transplant. All patients developed acute kidney injury and/or hepatic dysfunction before transplantation; however, none required dialysis or continuous renal replacement therapy. As expected, all 3 had high lung allocation scores, ranging from 89.9 to 90.6.

**Table 2:** Patient Characteristics

Patient #	Age	Dx	LAS	Indication for ECMO	Pulmonary hypertension	Preop ECMO	Preop ECMO days	Postop ECMO	Postop ECMO days	Kidney function pre-Tx	Liver function pre-Tx	Left graft ischemia time	Right graft ischemia time	Blood transfusion
1	62	ILD	90.6	Hypoxia, RV dysfunction	Yes	VV	9	cVA	2	Preserved kidney function	Hepatic dysfunction	3 hours 22 minutes	4 hours 25 minutes	4 U PRBC
2	61	ILD	89.9	Hypoxia, BiV dysfunction	No	fVA	5	cVA	2	AKI (Cr 1.55 up from 1.09 pre-Tx)	No hepatic dysfunction	4 hours 31 minutes	5 hours 52 minutes	9 U PRBC, 2 U PLT, 4 FFP
3	49	RA-ILD	90.2	Hypoxia, RV dysfunction	No	fVA to cVA	5	cVA	8	AKI (Cr 1.40 up from 0.89 pre-Tx)	Hepatic dysfunction	3 hours 1 minute	4 hours 33 minutes	15 U PRBC, 2 PLT, 2 FFP

Abbreviations: AKI, acute kidney injury; BiV, biventricle; Cr, creatinine; cVA, central venoarterial (right atrial to aorta); Dx, diagnosis; ECMO, extracorporeal membrane oxygenation; FFP, fresh frozen plasma; fVA, femoral venoarterial; ILD, interstitial lung disease; LAS, lung allocation score; PLT, platelets; postop, postoperative; PRBC, packed red blood cell; preop, preoperative; pre-Tx, pretransplant; RA-ILD, rheumatoid arthritis associated ILD; RV, right ventricle; U, unit; VA, venoarterial; VV, venovenous.

**Figure 1:**
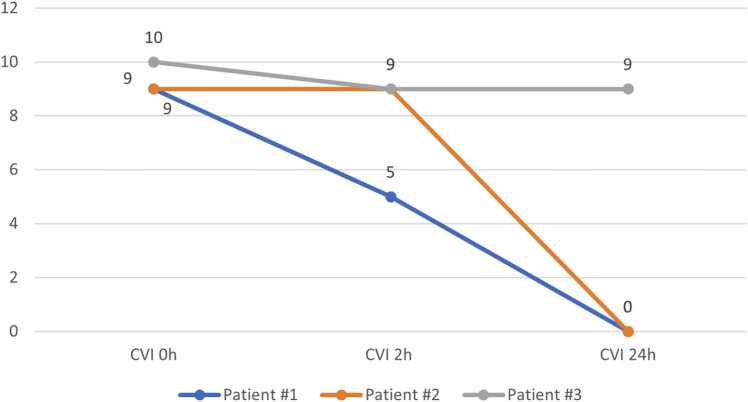
Cumulative vasopressor index within 24 hours after hydroxocobalamin administration. CVI, cumulative vasopressor index.

They were successfully bridged to double LTx via a clamshell approach. Intraoperatively, the right internal jugular VV ECMO or fVA ECMO was transitioned to central VA (cVA) ECMO to support the severely dilated and struggling right ventricle (RV). Post-transplant, the RV function remained significantly depressed, and the patients experienced severe refractory vasoplegia despite maintaining cVA ECMO support as a bridge to recovery and administration of multiple vasopressors. Consequently, they were treated with hydroxocobalamin in the intensive care unit. Pre- and post-transplant ECMO durations were 9, 5, 5 and 2, 2, 8 days, respectively. At baseline, before administration of hydroxocobalamin, all 3 were on norepinephrine, epinephrine, and vasopressin with CVI of 9 (patient #1), 9 (patient #2), and 10 (patient #3), respectively (Figure 1). Other vasoactive agents, such as methylene blue or angiotensin-II, were not used. Each patient received a standard pulse dose steroid wean as part of the triple-drug maintenance immunosuppression regimen, with 1 patient (patient #3) also receiving stress dose steroids. Vasopressor requirements of the 3 subjects are presented in Table 3.

**Table 3:** Mean Arterial Pressure at Time of Hydroxocobalamin Order and Vasopressor Use Within 24 Hours

Patient #	MAP at time of order	Number of Cyanokit doses	Vasopressor dose				CVI			Lactate	
			Name	0 h	2 h	24 h	0 h	2 h	24 h	0 h	24 h
1	61	1	Norepinephrine Epinephrine Vasopressin Dopamine	0.10 0.05 0.03 0	0.06 0.05 0 0	0 0 0 0	9	5	0	2.1	1.1
2	62	1	Norepinephrine Epinephrine Vasopressin Dopamine	0.10 0.05 0.02 0	0.10 0.05 0.04 0	0 0 0 0	9	9	0	8.5	1.7
3	62	7	Norepinephrine Epinephrine Vasopressin Dopamine	0.15 0.05 0.04 0	0.06 0.05 0.04 0	0.06 0.05 0.04 0	10	9	9	7.1	2.7

Abbreviations: CVI, cumulative vasopressor index; h, hours; MAP, mean arterial pressure (in mm Hg).

All doses are in mcg/kg/min except vasopressin, which is U/min. For patient #3, only the first 24 hours after hydroxocobalamin administration are presented in the table. See text for CVI data trends during subsequent days of hydroxocobalamin administration.

**Figure 2:**
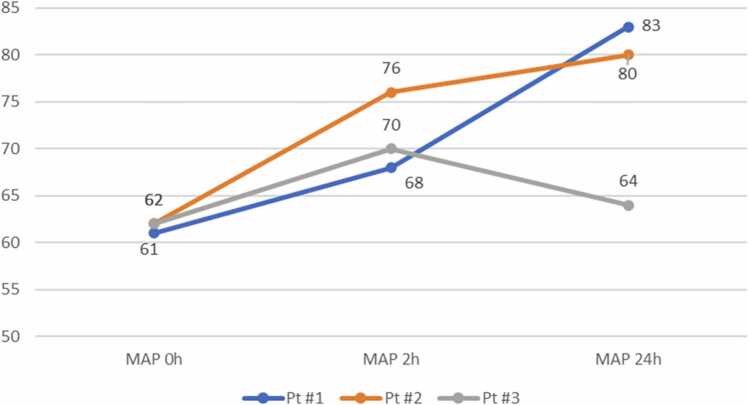
Mean arterial pressure within 24 hours after hydroxocobalamin administration. MAP, mean arterial pressure.

*Patient #1*: Two hours following the single intravenous (IV) bolus dose of hydroxocobalamin (5,000 mg infused over 15 minutes), there was a decrease in the CVI from 9 to 5 (reduction in the dose as well as the number of vasopressors), accompanied by an 11% increase in MAP. At the 24-hour mark, the CVI further decreased to 0, accompanied by a 36% increase in MAP ( Figures 1 and 2).

*Patient #2:* Two hours following a single IV bolus dose of hydroxocobalamin (5,000 mg infused over 15 minutes), the patient demonstrated a 17% increase in MAP. Despite this, the CVI remained at 9. At the 24-hour time-point, the MAP increased by 23% accompanied by a CVI decrease to 0 (Figures 1 and 2).

*Patient #3:* Two hours following a single IV bolus dose of hydroxocobalamin (5,000 mg bolus infused over 15 minutes) the CVI reduced from 10 to 9 while the MAP increased by 13%. At the 24-hour time-point, the CVI remained unchanged at 9 with MAP returning to prehydroxocobalamin baseline level (Figures 1 and 2). This patient received an additional 6 doses of hydroxocobalamin over the subsequent 4 days with similar trends. Notably, the drug was administered as an extended infusion over 6 hours as opposed to 15 minutes for the subsequent 6 doses. Stress dose steroids were administered on days 2 and 3 to support hemodynamics. By day 4, MAP increased by 15% with reduction in CVI from 9 to 0.

There were no allergic reactions and no episodes of severe hypertension in our case series. However, patient #3, who received a total of 7 doses of hydroxocobalamin, developed methemoglobinemia of 8.5%, which normalized after discontinuing the medication without further intervention. This patient also experienced mild, gradual hypertriglyceridemia up to 502 mg/dl over the course of several days while on a propofol infusion before hydroxocobalamin administration. After hydroxocobalamin administration, there was interference in the triglyceride lab assay, resulting in a sharp rise in triglyceride levels to over 1,000 ng/dl, despite propofol discontinuation for more than 16 hours. This laboratory interference was largely resolved within 4 days after discontinuation of hydroxocobalamin.

All 3 patients were discharged to home ambulating at 18, 26, and 65 days following LTx, respectively.

## Discussion

The use of hydroxocobalamin to treat vasopressor-resistant vasoplegia was first reported in cardiac surgery in 2014.^21^ Since then, several reports and case series have described its potential clinical benefits in cardiovascular surgery and liver transplantation. In a case series of 33 patients after cardiac surgery, 24 (72.7%) achieved hemodynamic improvement after 5-g hydroxocobalamin bolus administered over 15 minutes.^12^ Similarly, another case series of 10 patients with vasoplegia after left ventricular assist device placement showed that 6 (60%) improved after 5-gm bolus of hydroxocobalamin, administered over 15 minutes.^22^

The current case series is the first to report the use of hydroxocobalamin for treating vasoplegia following LTx. While prior studies have noted increases in MAP or decreases in vasopressor requirements after hydroxocobalamin administration in different patient populations,^17^ our case series is unique in its use of CVI to quantify the degree of vasopressor reduction. We believe this approach provides a more comprehensive and clinically relevant assessment of the treatment efficacy.

In the current study, we observed favorable effects at the 2-hour time-point in all 3 patients (patient #1: MAP+11%, CVI 9→5; patient #2: MAP+17%, CVI 9→9, and patient #3: MAP+13%, CVI 10→9). These findings suggest a potential benefit of hydroxocobalamin in treating vasopressors-resistant vasoplegia following LTx. This clinical benefit extended to the 24-hour mark in patient #1 (MAP+36%, CVI 5→0) and patient #2 (MAP+23%, CVI 9→0), while patient #3 MAP values returned to the prehydroxocobalamin baseline with unchanged CVI. Nonetheless, patient #3 displayed meaningful MAP improvement of 80 mm Hg given CVI of 0 on the fourth postoperative day, that is, after additional 6 doses of 5,000 mg hydroxocobalamin.

Overall, our results align with prior reports demonstrating improvements following either a single 5,000 mg bolus dose of hydroxocobalamin administered over 15 minutes^17^ or multiple doses.^23^ It is important to note, however, that there is currently no established guideline for the optimal dose or duration of hydroxocobalamin treatment of vasoplegia.

Only a few serious allergic reactions to hydroxocobalamin have been reported, including anaphylaxis, chest tightness, edema, urticaria, pruritus, dyspnea, and rash. Allergic reactions, such as angioneurotic edema, have also been reported in postmarketing experience.^24^, ^25^, ^26^, ^27^ None of our patients experienced such complications. However, all patients in our series demonstrated chromaturia, a well-known side effect of hydroxocobalamin.^17^, ^27^ Also, patient #3, who received multiple doses of hydroxocobalamin, developed methemoglobinemia, another known side effect. Methemoglobinemia can interfere with co-oximetry by artificially reducing SpO_2_, which is especially relevant in patients post-LTx. Additionally, patient #3 experienced hypertriglyceridemia despite discontinuation of propofol before hydroxocobalamin administration. This increase was later confirmed to be due to drug-laboratory test interference after confirmation with our laboratory personnel. This interference was resolved within 4 days following the cessation of hydroxocobalamin.

Acute kidney injury has been described with hydroxocobalamin use, with an incidence of 63% during the first 72 hours.^28^, ^29^, ^30^ The mechanism of renal injury is thought to be due to acute tubular necrosis with calcium oxalate crystal deposition. None of our patients developed kidney damage because of hydroxocobalamin use. One patient was on continuous renal replacement therapy for acute tubular necrosis following LTx but before hydroxocobalamin administration. This patient was discharged from the hospital on intermittent hemodialysis but ultimately kidney function recovered 32 days later.

Our 3 patients were critically ill and on ECMO both before and after LTx, which likely predisposed them to post-transplant vasoplegia in the absence of cardiac dysfunction, compared to patients not on ECMO.^9^, ^31^, ^32^ This small retrospective case series supports the safety and effectiveness of hydroxocobalamin for treating LTx recipients with vasopressor-resistant vasoplegia. However, further studies are needed to confirm these findings. Furthermore, patients should be monitored for methemoglobinemia and drug-laboratory interference following hydroxocobalamin administration.

## Author contributions

Anh Nguyen: Conceptualization, Methodology, Investigation, Formal analysis, Writing—Original Draft, Project administration. Rima Bouajram: Conceptualization, Methodology, Writing—Review and Editing. Marek Brzezinski: Conceptualization, Methodology, Writing—Review and Editing. Sahand Hassanipour: Project administration, Writing—Review and Editing. David Gordon: Investigation, Writing—Review and Editing. Binh Trinh: Resources, Writing—Review and Editing. Tobias Deuse: Resources, Writing—Review and Editing. Aida Venado: Resources, Writing—Review and Editing. Steve Hays: Resources, Writing—Review and Editing. Jonathan Singer: Resources, Writing—Review and Editing. Jasleen Kukreja: Conceptualization, Methodology, Supervision, Resources, Writing—Review and Editing.

## Disclosure statement

The authors have nothing to disclose.

Acknowledgments: None.
